# Impact of Intravenous Tranexamic Acid at Hospital Admission on Blood Transfusion Rates in Geriatric Extracapsular Hip Fracture Patients

**DOI:** 10.1155/aort/1177530

**Published:** 2025-12-28

**Authors:** Zachary Reynolds, Sarah Pirkle, Emily Williams, Zachary Langston, Alexander Hoffman, Kyle Adams, John D. (JD) Adams

**Affiliations:** ^1^ Department of Orthopedic Surgery, Prisma Health-Upstate, Greenville, South Carolina, USA; ^2^ University of South Carolina School of Medicine, Columbia, South Carolina, USA, musc.edu

## Abstract

**Purpose:**

Geriatric hip fractures often require allogenic blood transfusions, which increases the risk of transfusion related complications. Tranexamic acid (TXA) has been shown to have a positive effect on hip fractures, with the American Academy of Orthopedic Surgeons (AAOS) recommending its use; however, optimal dosing and timing has not been delineated in the literature. This study evaluates the effectiveness of early TXA administration in reducing transfusion rates in extracapsular geriatric hip fracture patients.

**Methods:**

From 2021 to 2023, a retrospective chart review compared geriatric fragility hip fracture (AO/OTA 31A) patients who received TXA at admission (1‐g IV at presentation and 1‐g IV three hours later) with those who did not. The primary outcome evaluated was allogenic blood transfusion rates. Patient demographics, hemoglobin levels during admission, hidden blood loss, length of hospital stay, and 90‐day perioperative complications were also recorded.

**Results:**

Among 168 patients, 102 received TXA and 66 did not. There was no statistically significant difference in allogenic transfusion rates between the groups (*p* = 0.27). Secondary outcomes, including hemoglobin/hematocrit levels, hidden blood loss, and length of hospital stay, were also similar. Thromboembolic event rates were comparable.

**Conclusion:**

Our findings align with recent literature that questions TXA’s effectiveness in reducing transfusion rates in this population. Early TXA administration may not adequately address hidden blood loss in geriatric hip fractures, possibly due to high comorbidity burden, pre‐existing anemia, and delayed surgery. Further research is necessary to explore alternative strategies for managing early blood loss and optimizing outcomes in this population.

## 1. Introduction

Each year, approximately 300,000 hip fractures occur in the United States, with projections reaching 500,000 by 2040 [1]. Research has increasingly focused on using tranexamic acid (TXA) to reduce blood loss and the need for allogenic blood transfusions in hip fracture patients. Transfusions carry risks such as transfusion reactions and increased postoperative infection risk. In addition, transfusions burden healthcare systems due to high costs and extended hospital stays [2–5]. Fragility hip fracture patients, often with multiple comorbidities, are more vulnerable to complications, functional decline, and mortality associated with acute blood loss anemia, making blood management critical in this population [4, 6].

In elective joint arthroplasty, TXA has been shown to safely reduce blood loss and transfusion rates, prompting further research on TXA use in hip fracture surgery [7–11]. TXA, whether administered intravenously or topically, has been shown to consistently reduce perioperative blood loss and the need for transfusions in hip fracture surgeries, with a favorable safety profile and no significant increase in thromboembolic complications [12–18]. However, this hip fracture literature is heterogeneous in both injury characteristics—such as femoral neck versus intertrochanteric fractures—and in the method of TXA administration, including intravenous (IV) and topical approaches. Specifically, some studies focus exclusively on intertrochanteric fractures [12, 18], others on femoral neck fractures [13], while several include a mixed population encompassing both injury types [14–17]. A systematic review by Farrow et al. examined seven randomized controlled trials (RCTs) on TXA use in hip fracture surgery, finding that while TXA reduced transfusion rates, variability in administration method (topical vs. IV) and timing underscored the need for further standardization [5].

Unrelated to the use of TXA, prior research has shown higher transfusion rates in extracapsular fractures compared with intracapsular fractures, most likely due to the large bone surface and the lack of containment of the fracture hematoma [19]. Although variability exists between fracture types, the American Academy of Orthopedic Surgeons (AAOS) has issued strong recommendations for TXA use in all geriatric hip fractures, not differentiating TXA use to specific fracture patterns [18, 20–22]. Although TXA use is strongly recommended, the optimal dosing regimen and timing has not been well defined. The variability of transfusion rates between extracapsular and intracapsular fractures prompted our investigation into whether early TXA administration at admission would reduce transfusion rates in extracapsular hip fractures. We hypothesized that TXA administered at emergency department presentation, with a second dose 3 h later, would reduce transfusion requirements in these patients.

## 2. Materials and Methods

After obtaining approval from Prisma Health’s Institutional Review Board, approval number 2077880, a single institution retrospective cohort study (Level III evidence) was performed to collect demographics and outcome data for patients with extracapsular, AO/OTA 31A, hip fractures. Inclusion criteria included patients over 65 years of age with fragility or low energy intertrochanteric hip fractures who had at least 90 days follow‐up data available for review. Exclusion criteria included admission hemoglobin value less than 7.0 g/dL or any concomitant injuries that would lead to increased confounding blood loss. Two cohorts of patients were identified: (1) patients who received TXA at admission and (2) those who did not.

### 2.1. TXA Protocol for Extracapsular Hip Fractures

The institutional protocol for administration of TXA was as follows: 1 g of IV TXA was given over 10 min at the time of presentation and diagnosis in the emergency department. This initial dose was followed by another 1 g of TXA 3 h later. Patients presenting with acute signs of a cerebrovascular incident or acute myocardial ischemia were not given TXA.

The primary outcome was allogeneic blood transfusions rates. Blood transfusion was performed when postoperative hemoglobin values were below 7.0 g/dL. Secondary outcomes included length of stay (LOS), lowest postoperative hemoglobin, blood transfusion volume, and 90‐day postoperative complications (including: surgical site infection, hematoma, ischemic cardiovascular event, cerebrovascular event, and deep venous thrombosis). The following patient demographics were recorded: age, gender, medical comorbidities (including history of diabetes, smoking, and anticoagulation use), mechanism of injury, and hemoglobin at admission.

### 2.2. Statistical Analysis

Continuous variables were compared between the TXA versus no TXA groups using *t*‐tests. Categorical variables were analyzed using Chi‐squared tests. Additionally, multivariate logistic regression was used to determine the impact of the TXA administration on blood transfusion rates, controlling for patient age, gender, ASA classification, anticoagulant use, time from admission to surgery, and AO/OTA fracture classification.

## 3. Results

Of the 168 patients included in the study, 102 patients received early TXA and 66 patients did not receive TXA. There were no significant differences in patient demographics and injury and fracture characteristics, including age, gender, hemoglobin at admission, ASA classification, AO/OTA fracture classification, anticoagulant use, and time from admission to surgery as seen in Table 1.

**Table 1:** Patient demographics including age, gender, hemoglobin at admission, ASA classification, anticoagulant use at admission, time from admission to surgery, and AO/OTA fracture classification.

Variable	TXA group (*n* = 102) Mean (±SD) or frequency (%)	No TXA group (*n* = 66) Mean (±SD) or frequency (%)	*p* value
Age at surgery	80.4 (±8.4)	80.7 (±9.5)	0.82

Gender	Male = 26 (25.5%)	Male = 21 (31.8%)	0.37
	Female = 76 (74.5%)	Female = 45 (68.2%)	

Hemoglobin	12.4 (±1.5)	12.3 (±1.9)	0.66

ASA classification	I = 0 (0%)	I = 0 (0%)	0.09
	II = 17 (16.7%)	II = 4 (6.1%)	
	III = 71 (69.6%)	III = 55 (83.3%)	
	IV = 14 (13.7%)	IV = 7 (10.6%)	

Anticoagulant use	Yes = 12 (11.8%)	Yes = 12 (18.2%)	0.35
	No = 90 (88.2%)	No = 54 (81.8%)	

Time from admission to surgery (h)	28.5 (±16.3)	28.4 (±15.9)	0.96

AO/OTA fracture classification	31A1 = 51 (50.0%)	31A1 = 38 (57.6%)	0.60
	31A2 = 31 (30.4%)	31A2 = 16 (24.2%)	
	31A3 = 20 (19.6%)	31A3 = 12 (18.2%)	

### 3.1. Primary and Secondary Outcomes

There was a total of 48 patients who underwent blood transfusion. No difference in transfusion rates was detected between the TXA group (25.5%) and no‐TXA group (33.3%) (*p* = 0.22). Based on the logistic regression model controlling for patient age, gender, ASA classification, anticoagulant use, time from admission to surgery, and AO/OTA fracture classification, TXA was not found to be a significant predictor of blood transfusion (adjusted odds ratio = 0.63; 95% CI = [0.30, 1.33]; ROC AUC = 0.73; and *p* = 0.23). In addition, no significant differences were found in any of the secondary outcomes including lowest postoperative hemoglobin, blood transfusion volume (Table 2), LOS, or 90‐day postoperative complications including cerebrovascular events, deep vein thrombosis, or ischemic cardiovascular events.

**Table 2:** Results of primary outcome and allogenic blood transfusion rate, as well as secondary outcomes: lowest hemoglobin and blood transfusion volume.

Variable	TXA group (*n* = 102) Mean (±SD) or frequency (%)	No TXA group (*n* = 66) Mean (±SD) or frequency (%)	*p* value
Blood transfusion	No = 76 (74.5%)	No = 44 (66.7%)	0.27
	Yes = 26 (25.5%)	Yes = 22 (33.3%)	

Lowest hemoglobin	8.5 (±1.6)	8.3 (±1.7)	0.42

Blood transfusion volume (mL)	586.7 (±455)	605.5 (±277)	0.87

## 4. Discussion

In this study, early administration of TXA, compared with no TXA, did not show a significant effect on transfusion rates in patients with extracapsular hip fractures. Most prior studies on this topic have been retrospective, with sample sizes ranging from 40 to 183 patients, yielding variable results similar to those observed in TXA studies on elective arthroplasty. Previous studies specific to hip fractures were often not standardized by fracture type, dosage, or timing of TXA administration.

Contrary to our findings, Moran et al. retrospectively evaluated all hip fractures (both extracapsular and intracapsular) undergoing surgery and concluded that TXA given at admission reduced transfusion rates compared with perioperative TXA alone [23]. However, closer examination of their results revealed that this difference was mainly seen in the intracapsular group, as patients who underwent intramedullary nailing (IMN) had similar transfusion rates (27%) to those who received no TXA (29%). Another study, which followed a protocol administering TXA at four different time points (within 8 h of injury, 1 h after the first dose, at incision, and 3 h postoperatively), found a significant reduction in transfusion rates compared to no TXA [24]. These studies indicate that TXA likely plays a role in managing blood loss in geriatric hip fracture patients, but the precise fractures and optimal timing for TXA administration remain unclear.

In our effort to isolate the effects of TXA in a specific fracture type and administration timing, we found that early TXA administration after admission for extracapsular fractures does not appear to influence transfusion rates.

A recent, large RCT by Owen et al. evaluated early TXA administration for extracapsular hip fractures and, consistent with our results, found no difference in transfusion rates between patients receiving early TXA and those who did not [25]. The combination of our results with this high‐quality RCT strengthens the conclusion that early TXA administration does not significantly affect transfusion rates in patients with extracapsular hip fractures.

Despite the lack of impact on transfusion rates, our study supports the safety profile of TXA, as we observed no significant differences in secondary outcomes such as surgical site infections, hematoma formation, ischemic cardiovascular events, cerebrovascular events, or deep venous thrombosis. This suggests that while TXA is safe for use in this population, its efficacy in reducing blood transfusion requirements, particularly when administered early, remains unproven.

The strengths of our study include a comprehensive evaluation of patient comorbidities and injury characteristics, with no significant differences identified between groups, along with strict exclusion criteria that allowed us to focus on a specific population: geriatric patients with isolated extracapsular hip fractures. However, the study’s small sample size may lend itself to a type 2 error. Therefore, this may limit the ability to draw definitive conclusions regarding transfusion rates from our study. However, while the small sample size is acknowledged as a weakness, recent high‐quality evidence supports the same conclusion. Additionally, the rationale for withholding TXA was not consistently documented in the medical record. Although a small subset of patients likely did not receive TXA due to appropriate clinical considerations—such as chronic kidney disease or a history of thromboembolic or cerebrovascular events—the absence of documentation suggests that most omissions were attributable to lapses in protocol adherence. Notably, the majority of patients in the no‐TXA cohort were treated prior to implementation of the institutional protocol, though a smaller number of omissions occurred after protocol initiation, further supporting inconsistent application rather than deliberate clinical decision‐making. This introduces the potential for selection bias, as patients in the no‐TXA cohort may not meaningfully differ from those who received TXA aside from variability in adherence to the institutional protocol. There is evidence that multidose TXA is most effective when administered within 72 h of injury, with studies demonstrating a reduction in perioperative hidden blood loss when this timing is achieved [26]. A limitation of our study is the lack of precise data on the interval between injury and TXA administration. As a surrogate, we used the time from hospital admission to TXA administration, which had a median of 4.5 h. For the majority of patients, this interval would reasonably fall within 72 h of injury; however, the exact timing relative to injury remains unknown. Of note, the absence of viscoelastic testing (e.g., LY30) in our study limited our ability to stratify patients by fibrinolytic phenotype. Recent work in total joint arthroplasty demonstrates that patients with fibrinolytic shutdown (FSD) do not respond to empiric TXA dosing, in contrast to nonshutdown (NFSD) patients receiving the same regimen [27, 28]. Future research should incorporate thromboelastography to resolve this heterogeneity in fibrinolytic phenotypes as well as evaluate optimal dosing regimen of TXA in patients with extracapsular hip fractures.

## 5. Conclusions

The importance of optimizing the medical management of geriatric patients with hip fractures cannot be overstated, given the high rates of morbidity and mortality in this vulnerable population [4, 29]. Our study hypothesized that early administration of TXA at admission would reduce “hidden blood loss” in extracapsular hip fractures, thereby decreasing the need for blood transfusions. However, our results disproved this hypothesis, demonstrating that preoperative IV TXA at hospital admission did not significantly impact allogenic blood transfusion rates in this patient group. Further research delineating the effects of TXA on specific fracture types and optimal timing of TXA is needed.

## Disclosure

This study data have previously been published in the form of a poster presentation at a local conference [30]. https://sc.edu/about/signature_events/discover_uofsc/find_presenter/?t=fmt=cat=sctn=plname=Langstonpcamp=pdept=req=lcomm=mlname=mcamp=mdept=subsearch=Search.

## Conflicts of Interest

John D. (JD) Adams, Jr. discloses the following affiliations: Arthrex, S&N, AAOS OKU Committee Member, Associate Editor—The Journal of Knee Surgery. The other authors declare no conflicts of interest.

## Funding

The authors declare that no funds, grants, or other support were received during the preparation of this manuscript.

## Data Availability

The data that support the findings of this study are available from the corresponding author upon reasonable request.
